# Novel Magnesium Elektron21-AlN Nanocomposites Produced by Ultrasound-Assisted Casting; Microstructure, Thermal and Electrical Conductivity

**DOI:** 10.3390/ma11010027

**Published:** 2017-12-26

**Authors:** Abdollah Saboori, Elisa Padovano, Matteo Pavese, Claudio Badini

**Affiliations:** Department of Applied Science and Technology (DISAT), Politecnico di Torino, Corso Duca Degli Abruzzi 24, 10129 Torino, Italy; Elisa.padovano@polito.it (E.P.); Matteo.pavese@polito.it (M.P.); Claudio.badini@polito.it (C.B.)

**Keywords:** nanocomposite, Elektron21, thermal conductivity, electrical conductivity

## Abstract

In the current work, a novel magnesium alloy Elektron21 reinforced by ceramic AlN nanoparticles were produced by an ultrasound-assisted casting. The fabricated nanocomposites were investigated to evaluate their microstructure, hardness, physical, thermal and electrical conductivity. The microstructural evolutions show that a uniform dispersion of the ceramic particles within the matrix can be achieved by employing the ultrasound-assisted stirring. However, some nanoparticles were found to be pushed by the solidification front. According to the Vickers hardness results, the addition of AlN nanoparticles results in a slight improvement of the mechanical properties of the nanocomposites. What is surprising is that both electrical and thermal conductivity of the nanocomposite were improved significantly as a consequence of AlN addition. This improvement in the conductivity characteristics of the nanocomposite is mainly corresponding to the structural effect of nanoparticles within the matrix.

## 1. Introduction

One of the solutions for reduction of fuel consumption and greenhouse emissions in the automotive and aeronautic industries is light weighting that can address the present and future challenges [1,2,3]. Among all the metals, magnesium could gain widespread attention in these industries owing to its strength/ductility ratio and low density [4,5]. Nowadays, Mg alloys with elements of rare earth (RE), in particular, Elektron21 (El21), have been introduced in the aerospace applications because of its improved corrosion resistance and production costs. However, not all applications allow working with El21 and also improvement of mechanical properties of complex as-cast parts by heat treatment is most of the time limited or impossible [6]. Good castability, fine grains and clean microstructure are the main factors for designing a lightweight castable alloy that can be full filled with the homogeneous addition of nanoparticles in the molten alloy. Besides the mechanical strength at high temperatures, thermal properties such as specific heat capacity and thermal conductivity are very important for high-temperature applications [4,7]. Thus, it is necessary for the metallurgical industries to determine these properties precisely. So far, however, there has been little data about the thermal properties of El21 and no data regarding the El21 nanocomposites. 

The aim of this paper is to explore the effect of ultrasound-assisted stirring on the dispersion of the AlN nanoparticles, to investigate the influence of nanoparticles on the Vickers hardness, electrical and thermal conductivity of the novel El21-AlN nanocomposites for the first time.

## 2. Materials and Methods

Commercial El21 (composition in wt.%: Mg-2.8Nd-1.2Gd-0.4Zr-0.3Zn was melted and kept at 720 °C for 1 h and then poured into a preheated cylindrical mould. Thereafter, the spherical AlN nanoparticles (20–30 nm) with 20% pure Al as an impurity were added into the melt, stirred and power ultrasound was additionally applied for 6 min at 0.3 kW under a flux of protective gas. After sonication the mould was could down in a water bath. 

Hardness measurements were undertaken with a load of 5 kg and the dwell time was 15 s. A Field-Emission Scanning Electron Microscope (FESEM; Merlin-Zeiss; Munich, Germany) equipped with an energy-dispersive X-ray spectrometer (EDS; Munich, Germany) was employed for microstructural evaluations. Laser-flash method was used to measure the thermal diffusivity, by using a FLASHLINETM apparatus (Anter corporations, Pittsburgh, PA, USA). The electrical conductivity of El21 and its nanocomposites was measured by means of an eddy current electrical conductivity meter. All the results of electrical conductivity, thermal conductivity and Vickers hardness are taken from an average of three measurements. 

## 3. Results and Discussion

**Figure 1:**
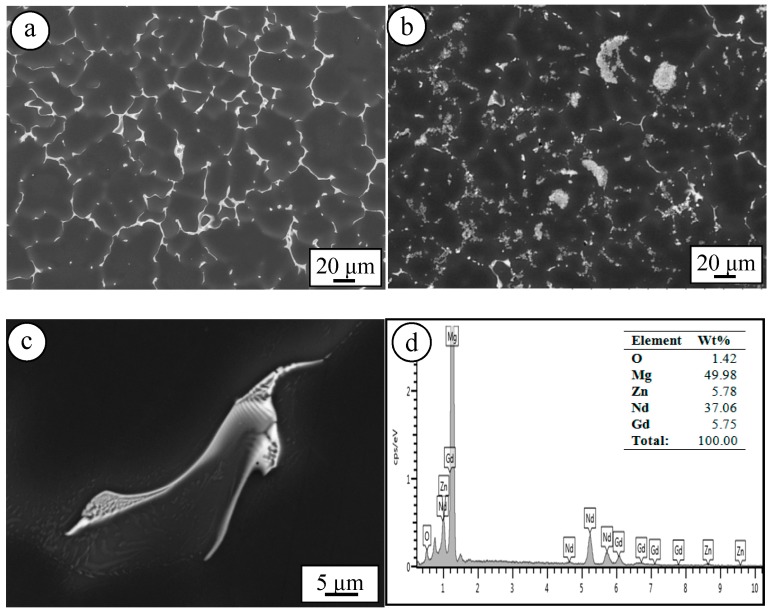
Microstructure of as-cast (**a**) El21; (**b**) El21-2%AlN; (**c**,**d**) El21 and its corresponding EDS.

The as-cast El21 alloy which is shown in Figure 1a is characterized by α-Mg solid solution and Mg_3_(Nd,Gd) eutectic phase on the grain boundaries. Indeed, Mg_3_(Nd,Gd) eutectic phase is a modification of Mg_3_Nd phase where the Nd is partially replaced by Gd without modifying the crystal structure. Since the difference between the atomic radii of Nd and Gd is negligible this substitution does not destroy the crystal structure [7]. The EDS analysis of the Mg_3_(Nd,Gd) eutectic phase confirms the simultaneous presence of Gd and Nd in the eutectic phase (Figure 1c,d). The average grain measurement which is undertaken by image analysis software has shown that the addition of AlN nanoparticles results in a slight gain refinement. Indeed, the average grain size El21 which is 80.1 μm is reduced to 74.1 and 72.8 μm for El21-1%AlN and El21-2%AlN, respectively. As can be seen in Figure 1b, which is the microstructure of El21-2%AlN despite the presence of some agglomerates, the dispersion of AlN nanoparticles seems to be rather homogeneous.

**Figure 2:**
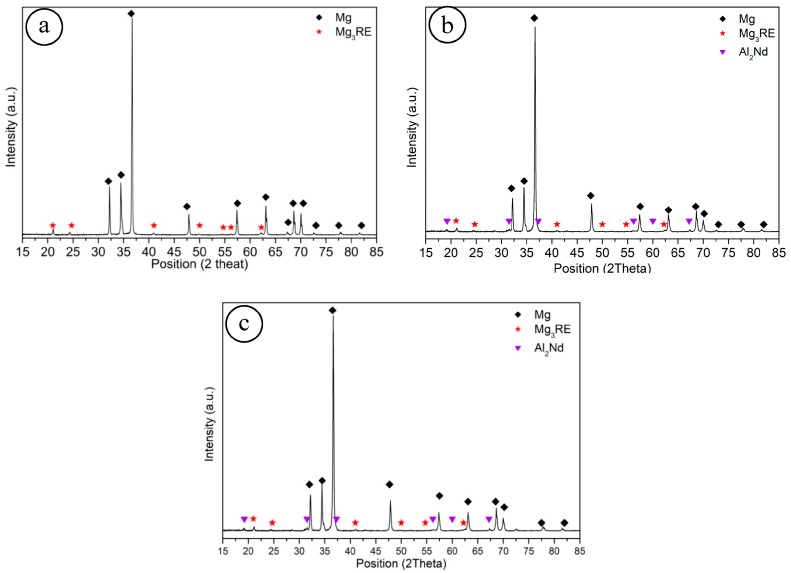
X-ray diffraction patterns of (**a**) El21; (**b**) El21-1%AlN; (**c**) El21-2%AlN in as-cast condition.

X-ray diffraction patterns of as-cast EL21 which is shown in Figure 2a identified the main peaks corresponding to Mg (the matrix phase). Moreover, the peaks related to the Mg_3_RE phase as the main intermetallic phase were detected. Surprisingly, in as-cast condition, the El21 alloy already displays a high density of intermetallic precipitates. However, in the X-ray patterns of as-cast El-1%AlN and as-cast El-2%AlN nanocomposite which are shown in Figure 2b,c the main peaks corresponding to Al_2_Nd were detected beside the other peaks (Mg and Mg_3_RE). This means that part of Al introduced as an impurity with AlN particles reacts with Nd and forms Al_2_Nd.

**Figure 3:**
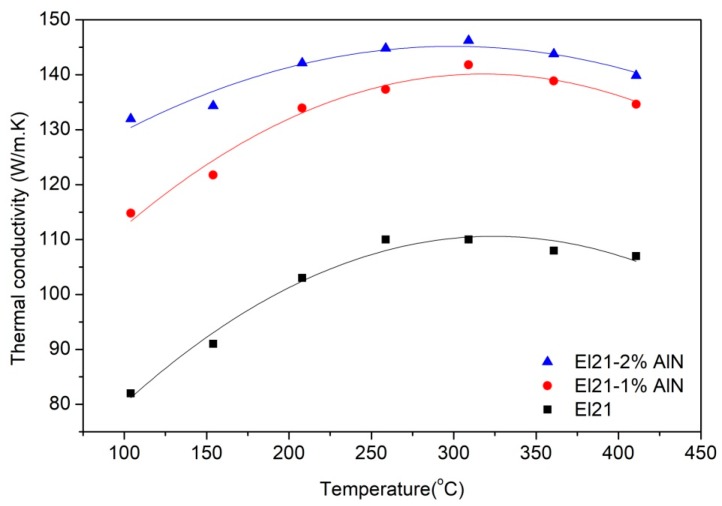
Temperature dependence of the Thermal conductivity of El21, El21-1%AlN and El21-2%AlN in as-cast condition.

Temperature dependence of the Thermal conductivity of El21, El21-1%AlN and El21-2%AlN in as-cast condition is shown in Figure 3. In these novel El21-AlN nanocomposites, the thermal conductivity of the nanocomposites was improved significantly as a function of AlN content. Indeed, this noticeable improvement in the nanocomposites can be related to the two main phenomena. The first reason is related to the microstructural effect of AlN nanoparticles which contained some Al as an impurity. Generally speaking, Mg is well-known because of its high thermal conductivity and high efficiency of heat dissipation [8]. It is found that in multicomponent alloys the solute elements in the alloy act as scattering centers of phonon contribution to thermal transfer [9]. In this work, as a matter of fact, as shown by XRD patterns Al particles which were inserted in the nanocomposites reacted with Nd which is a solute element and forms Al_2_Nd. It implies that this reaction results in the reduction of scattering point and consequently improvement of the thermal conductivity of the nanocomposite with respect to the El21. Indeed, these findings are in line with the previous works [7,9].

Kielbus et al. have investigated the effect of solute elements on the thermal conductivity of Mg [7]. For this reason, they compared the thermal conductivity of EL21 and WE54 (Mg-Y-Nd) before and after the solution treatment. In both cases, the thermal conductivity of alloys was very low because of their higher solute element content in the α-Mg matrix. In addition, they have shown that the thermal conductivity of El21 was higher than WE54 because of the lower amount of elements and lower dissolubility of Gd (in comparison with Gd) in solid solution. The second reason for thermal conductivity improvement could be as a consequence of the reaction bonding between the nanoparticle and the matrix that results in the thermal conductivity improvements. Indeed, it is reported that Zr atoms which are a parent in the matrix can be incorporated in the AlN surface and accordingly ZrN forms and some Al released [10]. This released Al can also consume the Nd element a solute atom and form Al_2_Nd (as shown earlier by XRD). These Consecutive reactions improve the thermal conductivity of El21-AlN nanocomposites through two mechanisms; (i) formation a reaction bonding between the matrix and reinforcement and (ii) consumption of a solute element which has a noticeable effect on thermal conductivity of El21 alloy. 

Table 1 shows the Vickers hardness and electrical conductivity results of El21 and El21-AlN nanocomposites. As can be seen in this table, the Vickers hardness of El21-AlN nanocomposites improved slightly. This slight improvement in Vickers hardness of nanocomposites shows that the incorporation of AlN nanoparticles which does not result in the grain refinement, cannot improve the Vickers hardness of nanocomposites. Nonetheless, this slight increase in the Vickers hardness of nanocomposite could be as a consequence of the incorporation of AlN nanoparticles. As a matter of fact, the nanoparticles within the matrix could hinder the dislocation motions and accordingly improves the hardness of the composite. The electrical conductivity results of El21 and its nanocomposites at the ambient temperature is presented in Table 1. 

It is evident that as same as the thermal conductivity of nanocomposites, their electrical conductivity is increased noticeably as a consequence of decreasing the scattering points of free electrons within the matrix through the solute element consuming. As discussed earlier some pure Al particles is introduced into the El21 and reacts with Nd which is a solute atoms and consequently reduced its content in solid solution and leads to the electrical conductivity improvement. 

## 4. Conclusions

Magnesium-based nanocomposites consist of Elektron21 as a matrix and AlN nanoparticles as reinforcement were successfully produced by ultrasound assisted casting technique. Microstructural evaluations show that the AlN nanoparticles were dispersed uniformly within the matrix. However, some AlN agglomeration has been detected after casting as a consequence of particle pushing during the solidification of nanocomposites. Moreover, it is found that the addition of AlN nanoparticles did results in a slight reduction in grain size and a slight increase in hardness with the addition of nanoparticles. The thermal and electrical conductivity results of nanocomposites show a significant improvement with respect to the El21 alloy. The XRD analysis confirms that the pure aluminium which is incorporated within the molten alloy as an impurity together with AlN nanoparticles and also released after the reaction between the Zr and AlN reacts with Nd and creates Al_2_Nd phase. This reaction between Al and Nd leads to the consumption of Nd which is an alloying element and reduce its amount in the solid solution. 

## Figures and Tables

**Table 1:** Vickers hardness and electrical conductivity of El21 and its composites at ambient temperature.

Material	EL21	El21-1%AlN	El21-2%AlN
Vickers hardness (HV_5_)	55 ± 2.1	60 ± 1.2	62 ± 1.1
Electrical conductivity at room temeprature (S/m)	1.34 × 10^7^	1.86 × 10^7^	2.06 × 10^7^
